# The Role of General and Specific Cognitive Abilities in Predicting Performance of Three Occupations: Evidence from Bifactor Models

**DOI:** 10.3390/jintelligence9030040

**Published:** 2021-08-17

**Authors:** Khalid ALMamari, Anne Traynor

**Affiliations:** Department of Educational Studies, Purdue University, West Lafayette, IN 47907, USA; atraynor@purdue.edu

**Keywords:** cognitive abilities, general ability, job performance, aviation, predictive validity, bifactor model

## Abstract

Cognitive abilities are related to job performance. However, there is less agreement about the relative contribution of general versus specific cognitive abilities to job performance. Similarly, it is not clear how cognitive abilities operate in the context of complex occupations. This study assessed the role of cognitive abilities on the performance of three aviation-related jobs: flying, navigation, and air battle management (ABM). Correlated-factor and bifactor models were used to draw a conclusion about the predictive relations between cognitive abilities and job performance. Overall, the importance of particular cognitive abilities tends to vary across the three occupations, and each occupation has different sets of essential abilities. Importantly, the interplay of general versus specific abilities is different across occupations, and some specific abilities also show substantial predictive power.

## 1. Introduction

The importance of general cognitive ability, *g*, as compared to specific abilities for job performance has been a subject of great debate (Kell and Lang 2018). Despite the assertion that cognitive abilities are among the best predictors of job performance, the controversy as to which ability or set of abilities plays a significant role in explaining variance in the criterion space of job performance has never ended. In this context, many studies have been and are being published to advocate the importance of some particular ability over other abilities for job performance prediction. The works of Schmidt and Hunter (1998, 2004) are examples of a strong line of research ascertaining that *g* is the most crucial ability for predicting occupational performance, whereas specific abilities do not explain much variance beyond *g*. Hunter (1986, p. 341) took an extreme position when he stated that “it is general cognitive ability and not specific cognitive aptitudes which predict job performance.” Schmidt (2002) argued that it is “not logically possible” to have a serious debate over the importance of general cognitive ability for job performance. In the same way, the “Not Much More Than g” series of Ree and his colleagues (Ree and Earles 1991, 1996; Ree et al. 1994) is a reflection of the same standpoint that views *g* as the best construct for the prediction of job performance. One implication of such a hypothesis is that the focus in selection procedures should be directed, to a large extent, to applicants’ scores of general ability (or IQ) and, to a much lesser extent, to their narrower ability scores.

Opposing this line of cognitive ability research, another direction has started to gain attention in recent years, emphasizing that specific abilities (e.g., verbal, quantitative, spatial) can also be significant components for predicting success in occupations, and their roles should not be ignored (e.g., Krumm et al. 2014; Lang and Kell 2020; Murphy 2017; Reeve et al. 2015; Schneider and Newman 2015; Wee et al. 2014). The idea of having one single trait, *g*, capable of fully capturing the individual differences in job performance might be problematic for applied industrial/organizational (I/O) psychology (Beier et al. 2019), particularly for selection and assessment purposes. Beier et al. (2019) noted that three challenges arise when relying solely on a *g* score: violation of legal frameworks in some organizations (e.g., not complying with job analysis), limitations of the information obtained from one single score, and the large majority–minority differences typically associated with *g* scores. Criticism was raised that research examining the prediction of job performance often takes *g* for granted, and other abilities are considered only for the sake of a little improvement (Ziegler and Peikert 2018). Stankov (2017) argued that the overemphasized “*g*” has hindered the study of broad and specific cognitive abilities and led to neglecting the first- and second-stratum factors in the Cattell–Horn–Carroll (CHC) model. Similarly, Murphy (2017) noted that studies stressing *g* measures over measures of specific abilities fail to consider the second-stratum abilities that can sometimes be more predictive for job performance than more global measures of general cognitive ability. He cautioned that the increasing publications overstressing the predictive role of *g* and underestimating the incremental contribution of specific abilities might have led to a premature decline in research on the roles of specific abilities in the workplace (Murphy 2017).

In contrast to the “Not Much More Than *g*” hypothesis, Kell and Lang (2017) maintained that specific abilities in some workplaces could be “More Important Than *g*.” The supporters of this contention believe that many of the findings that have devalued the significance of specific abilities in workplaces were due to limitations in the analytical procedures used in the assessment of predictive relations. The majority relied primarily on traditional regression analyses (e.g., hierarchical linear regression), which might not be the ideal analyses for making a firm conclusion about the relative importance of predictors. Although this family of statistical techniques is powerful in maximizing the prediction of a particular set of variables, they tend to provide an “unequal” opportunity for predictors to exhibit their potential power, especially when the multicollinearity among predictors is high (Tonidandel and LeBreton 2011). 

In hierarchical regression analyses, the frequently used method in incremental validity studies, a score of *g* (often the first unrotated principal component or composite score from a test battery), is entered first in the model, whereas specific abilities are added second in the model (e.g., Ree et al. 1994). Criterion scores (e.g., flying performance) are regressed first on scores of *g*, with scores of specific abilities (e.g., spatial ability, perceptual speed) entered in the second step of a hierarchical regression. The shared variance in this statistical design is always attributed to the influence of *g* because the model prioritizes predictors entered first into the hierarchical regression, regardless of specific-abilities variance shared with the criterion. Even the overlapping shared variance between *g* and specific abilities is counted as resulting from *g*. The only variance that is credited to other predictors in the model is the percentage that does not overlap with *g*. Such an analytical strategy is likely to leave little remaining variance in criterion scores that can be accounted for by specific abilities (Lang et al. 2010).

For that reason, many researchers have called for adapting other analytical procedures when attempting to establish whether specific abilities have incremental validity above and beyond that provided by *g*. Relative importance analysis (RIA) is one useful analytical procedure for investigating predictor–criteria relationships. Two variants of RIA have gained popularity in recent years: relative weight analysis (Johnson 2000) and dominance analysis (Azen and Budescu 2003). Both procedures have beenfound to produce similar results, although they differ in their computational and analytical foundations (Johnson 2000). The two procedures allow for a more accurate partitioning of variance in multiple regression, which leads to a better judgment of the effect of predictors on outcomes. RIA exhibits the impact each predictor has on the overall model, considering both its unique contribution and its contribution in the presence of other predictors (LeBreton et al. 2007). It decomposes the total predicted variance in a criterion into that which should be attributed to each individual predictor, even when the predictors are strongly correlated with one another. These analyses, however, are not meant to be a replacement for regression analyses but rather an informative supplement fostering the understanding of the role played by each predictor in a regression equation (Tonidandel and LeBreton 2011). Although relative weight and dominance analysis are very useful techniques for assessing the relative importance of predictors in a model, neither is as powerful as multiple regression in maximizing the prediction of the criterion variable.

A bifactor model, or nested-factor model, is another useful approach to help improve our understanding of the interplay of predictors. Although this model was introduced many decades ago (Holzinger and Swineford 1937), its use as a predictive model for associations between predictors (e.g., cognitive abilities) and outcome criteria (e.g., job performance) has been revived only recently. In a bifactor model, *g* is modeled similarly to specific abilities as a lower-order factor but differently in that it has paths to all (or the majority) of the indicators. Studies comparing bifactor models with higher-order models have showed that bifactor models tend to produce a better fit than higher-order models (e.g., Cucina and Byle 2017; Morgan et al. 2015). The bifactor model’s unique specification allows for an effective partitioning of variance among observed variables and enables a clear separation of domain-general from domain-specific effects (Reise 2012; Zhang et al. 2021). The *g* effect can thus be disentangled from specific-ability effects, and their contributions to a criterion can be assessed using latent multiple regression models underlying the SEM framework. The readily built-in orthogonalization feature in this model makes it appropriate for investigations that seek a complete distinction between the effects of general and specific factors (e.g., Gignac 2008). 

Alternatively, Coyle (2014) advocated the use of an analytic approach through which relations are tested via the non-*g* residuals of tests produced from a higher-order factor model (i.e., a hierarchical structure involving three conceptual levels: *g* at the top, ability group factors at the second level, and specific abilities at the lowest level represented by observed test scores). He argued that this approach is the most promising approach in the study of human intelligence (Coyle 2014). In these SEM models, the residuals of specific abilities are allowed to be correlated with performance measures to partial out the effect of *g*, thus providing a purer estimate for specific-abilities effects on performance. Relations that were examined with the non-*g* residuals of tests showed that specific abilities could have equal or even higher importance than *g* in predicting outcomes. Contrary to the primacy of *g* hypothesis, Coyle (2018) found significant incremental validity for several specific abilities on the SAT, ACT, and Preliminary SAT tests above *g* validity for the prediction of different criteria, often with substantial effect sizes (βs ≈ 0.30). This method has seen increased use and has assisted in determining the relative role of specific constructs beyond the validity obtained by the *g* factor (e.g., Benson et al. 2016; Berkowitz and Stern 2018; Wee 2018).

In addition to the influence of statistical analyses on the results concluded from predictive validity research, there are other factors that can determine whether or not specific abilities are important predictors for job performance. The cognitive-ability–job-performance compatibility principle (Schneider and Newman 2015) is one factor that needs to be considered in such investigations and is believed to be one possible reason biasing against specific abilities. The center point here is the necessity to make a reasonable alignment between predictors and criteria such that a general predictor is matched with a general criterion and specific predictors are matched with specific criteria (Wee 2018). More precisely, as indicator variables for a predictor and criterion have similar cognitive requirements and are equally weighted in the predictor and criterion, the strength of the predictive relationship is expected to increase (Krumm et al. 2014, citing Brunswik’s (1956) lens model). 

Moreover, the job performance dimension is another aspect to take into account when designing a criterion-related validity study. Drasgow (2012) argued that expanding the criterion space to include other criteria than training performance and overall job performance (e.g., contextual job performance, counterproductive work behaviors, and attrition) enables a better understanding of the individual differences that predict behavior in the workplace. Derived from an integrative synthesis of the literature, Campbell and his colleagues (e.g., Campbell et al. 2001; Campbell and Wiernik 2015) proposed an eight-factor model representing the primary dimensions of performance in a work role. A hierarchically organized structure, similar to an intelligence model, was also suggested for job performance, where indicators from different performance domains cluster into a few group factors of broad performance (or compound performance dimensions) and the highest order factor of performance is at the vertex of the model (Ones et al. 2017). Hence, a more thoughtful plan in the design of a validation study, particularly related to the selection of criteria, can have an impact on the results and conclusions determined about the true relations between predictor and outcome variables. 

Another factor that can be highlighted in ability–performance research is the overuse of correction (LeBreton et al. 2014). The compelling results showing the negligible role of specific abilities relative to the predominant role of general ability for predicting job performance may be due, in part, to studies’ reliance on correlations that have undergone several corrections for range restriction, measurement error, or dichotomization. Although the correction of observed correlations is a recommended strategy to produce more accurate estimates of ability–performance relationships, it may have precluded critical evaluations and possible refinement of the interplay of general and specific cognitive abilities in predicting job performance. It might have also hindered scholarly understanding and appreciation of the possible role of specific abilities as a worthy predictor for future work outcomes. Thus, in this study, we applied uncorrected data (i.e., observed correlations) to establish more clearly the relative contribution of cognitive abilities for predicting job performance, free from the possible influence of correlation correction. 

The bright side of this long-lived scientific debate, however, is that it has stimulated dynamic research in both directions, which is certainly advantageous for the advancement of related sciences. Some journals have devoted special issues debating the relative value of cognitive abilities for performance outcomes. As an example, a special issue of *Human Performance* discussed the role of general mental ability in I/O psychology (Viswesvaran and Ones 2002). Equally, a recent special issue of *Journal of Intelligence* focused on this great debate in seven articles (Kell and Lang 2018) in an attempt to motivate reconsideration of specific abilities in the workplace. Some of these articles offered analytical strategies that can be used as an alternative to the traditional statistical analysis to disclose the determinants of job performance more accurately (e.g., Coyle 2018; Eid et al. 2018; Ziegler and Peikert 2018). Of interest, this debate on the relative role of general versus specific abilities has transferred from educational and workplace settings to other life domains. Some forms of this debate can now be found in studies of wages (Ganzach and Patel 2018), players of the National Football League (Lyons et al. 2009), happiness (Blasco-Belled et al. 2020), triangular love (Van Buskirk 2018), humor production ability (Christensen et al. 2018), music training (Silvia et al. 2016), and piano skill acquisition (Burgoyne et al. 2019).

The present study revolved around this context—the debate on whether it is general ability or specific abilities that contribute most to the prediction of job performance. More specifically, we assessed the role of five specific abilities (verbal, quantitative, spatial, perceptual speed, and aviation-related acquired knowledge), as well as general ability, in predicting performance in three military aviation-related occupations: flying, navigation, and air battle management (ABM). Given the nature of the three occupations, the selectees to these jobs are typically of high cognitive aptitude and they achieve high scores in many selection requirements such as scholastic, personality, physical, and medical examinations. Hence, there is more opportunity for cognitive abilities to demonstrate their roles and influence in the individuals’ performance. In this study, we aimed to understand how influential certain cognitive abilities are in different aviation occupations, and how the occupational patterns may vary. 

The examination of relationships relied primarily on a bifactor modeling approach as a suitable alternative statistical approach. In this study, we sought to examine latent relationships between cognitive abilities and job performances, which can be accomplished appropriately through SEM procedures. We were interested in capturing the latent constructs of cognitive abilities and related them to latent (or observed) job performance. SEM can be a sound method for this particular goal as compared to other alternative analyses more suitable for the assessment of scores at the observed, lower abstract level (e.g., Oh et al. 2004; Glutting et al. 2006). Through SEM application, we can also overcome the concerns raised around the hierarchical regression analysis. Given the goals pursued by the current investigation, a bifactor SEM model provides an efficient tool to disentangle the effect on criteria due to the general factor from the effects due to the specific-ability factors, with several equations and parameters tested simultaneously. Every ability factor, including the *g* factor, will have a path (i.e., regression) coefficient showing its effect on performance criteria, controlling for other abilities in the model. Thus, the unique contribution of every ability to the candidates’ outcomes in the three aviation jobs can be estimated. The two main research questions investigated in the present study were as follows: (1) *How do the predictive relations between cognitive abilities and job performance vary across the three occupations (flying, navigation, air battle management (ABM))?* (2) *Is there any incremental validity of the specific group factors of the abilities above that obtained from the g factor in any of the three occupations (flying, navigation, ABM)?*

## 2. Materials and Methods

### 2.1. Subjects

Data for pilots and navigators were obtained from the uncorrected correlation matrices reported in Olea and Ree’s (1994) study, while the data for air battle managers were obtained from the uncorrected correlation matrix reported in Carretta’s (2008) technical report. The three correlation matrices are presented in Appendix A. The pilot sample consisted of 1867 undergraduate pilot training (UPT) students. The navigator sample included 957 undergraduate navigator training (UNT) students. The subjects of both samples were tested for officer selection between 1981 and 1985, and all possessed baccalaureate degrees when they began training. The time elapsed between cognitive testing and criterion data collection was between 13 months and four years. The air battle manager sample consisted of 680 undergraduate air battle manager (UABM) training students who had been tested for officer selection between 1999 and 2007. The Air Force Officer Qualification Test (AFOQT) was the primary selection tool used in qualifying the subjects of all three samples for the officer training programs. In addition, the selected applicants had to meet other selection requirements (e.g., academic, fitness, medical, moral, and physical standards). 

### 2.2. Measures

#### 2.2.1. Cognitive Abilities 

The cognitive ability test scores were obtained from the AFOQT testing during selection. The AFOQT is a multiple-aptitude test battery used by the U.S. Air Force (USAF) to select officer candidates and qualify them for different USAF jobs, including aviation professions. The AFOQT scores used in this study came from two different forms: Form O (Carretta and Ree 1996), which was administered to the pilot and navigator samples (16 subtests), and Form S (Drasgow et al. 2010), which was administered to air battle managers (11 subtests). Although the two forms consisted of a different number of subtests, the 11 subtests in Form S had been retained from earlier AFOQT versions (Form O, P, and Q). Thus, the 11 subtests in both versions are equivalent and represent similar contents and constructs. Ten of the 11 subtests were used to test factor-analytic AFOQT models for each of the three data sets. 

Consistent with the five-factor model previously proposed for the AFOQT (Carretta and Ree 1996; Carretta et al. 2016; Drasgow et al. 2010), confirmatory factor analysis (CFA) measurement models representing five group factors were specified. Specifically, two subtests were selected as indicators for each of the five domains of cognitive abilities: Verbal Analogies (VA) and Word Knowledge (WK) for verbal ability, Arithmetic Reasoning (AR) and Math Knowledge (MK) for quantitative ability, Rotated Blocks (RB) and Hidden Figures (HF) for spatial ability, Table Reading (TR) and Block Counting (BC) for perceptual speed, and Instrument Comprehension (IR) and Aviation Information (AI) for the domain-specific ability of aviation-related acquired knowledge. Although the AFOQT test battery was designed to measure multiple cognitive domains, and its composite scores have been shown to be multidimensional, it should be noted that the battery was not designed to maximize predictive value of the underlying specific factors. Thus, to some degree, our secondary analysis of the subtest scores’ correlations used these data for a different purpose than was originally intended.

#### 2.2.2. Performance Measures 

Olea and Ree (1994) reported six outcome criteria for both pilot and navigator samples. From these measures, three for each sample that covered the performance space adequately were selected to be used in the current study. The selected criteria for flying performance were Pass/Fail training, Phase 2 average rating, and Phase 3 average rating. Pass/Fail training indicated the final training outcome and the overall performance of students academically, professionally, and even ethically. The ratings from Phase 2 and Phase 3 of training were cumulative grades covering different aspects of flying and airmanship, such as flying performance in daily flight and check flight, commanders’ ratings, and several written tests on aeronautical topics. The selected criteria for navigation performance were Pass/Fail training, day check flight, and night check flight. Day and night check flights represented actual work samples for students, which covered essential navigation skills such as stellar observations, sun plotting, and real flight missions. The performance criteria in ABM training were one overall index given to the students, representing an average final score on several written tests taken during the training course (Carretta 2008).

### 2.3. Analytic Plan

**Figure 1:**
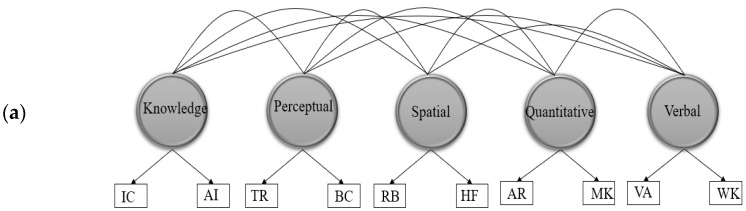

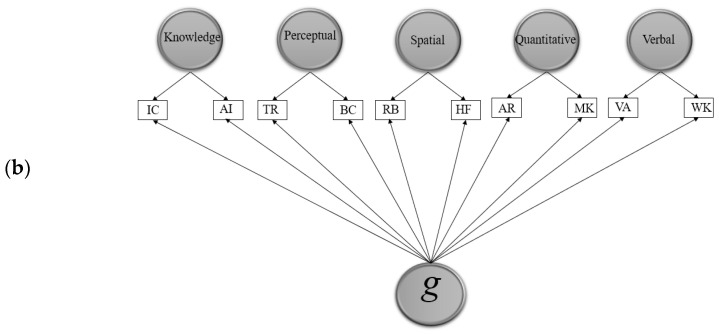
Representation of the AFOQT correlated-factor (**a**) and bifactor (**b**) models. Note: VA = Verbal Analogies; WK = Word Knowledge; AR = Arithmetic Reasoning; MK = Math Knowledge; RB = Rotated Blocks; HF = Hidden Figures; TR = Table Reading; BC = Block Counting; IR = Instrument Comprehension; AI = Aviation Information.

Through a SEM framework, each of the three samples underwent a separate analysis but with similar analytic procedures. Only one difference may be highlighted here concerning the ABM sample criterion’s modeling. Due to the single criterion available for this sample, we modeled it as an observed variable, while the criteria in the other two samples were modeled as latent variables constructed with three different observed scores. We planned the following analytic procedure for each of the three samples. First, we tested a CFA measurement model comprising five specific cognitive abilities. Two models were assessed: a correlated-factor model and a bifactor model. Figure 1a,b displays the two CFA models specified at this stage. This test was necessary to confirm the plausibility of the suggested five-factor model and to ensure the adequacy of fit before moving to the SEM stage of analyses. 

Second, we specified a combined correlated-factor model containing the ability factors and the criterion factor (or the observed score in the case of ABM). This model was estimated to verify the existence of relationships between the ability factors and performance criteria. Third, we specified bifactor SEM models to estimate the effects of the five specific cognitive abilities, as well as the *g* factor, on the performance measures. Bifactor modeling was found to be an effective technique for assessing the predictive role of general and specific abilities. For model identification purposes, the loadings of the two test score indicators of each of the five specific factors (verbal, quantitative, spatial, perceptual speed, acquired knowledge) were constrained to be equal (e.g., Green and Yang 2018). This was applied to both types of models, correlated-factor and bifactor. Under this setting, the single loading value estimated might be interpreted, loosely, as an average loading for that indicator pair. 

All CFA and SEM models were estimated using maximum likelihood (ML). Models’ fit was assessed according to several goodness-of-fit indices, including comparative fit index (CFI), root mean square error of approximation (RMSEA), and standardized root mean square residual (SRMR). As recommended by Hu and Bentler (1999), good fit between the hypothesized model and the observed data requires a value close to 0.95 for CFA, although any value over 0.90 is considered acceptable. Similarly, RMSEA and SRMR values close to 0.06 indicate good fit, although values as high as 0.08 are considered acceptable. For chi-square (χ^2^), due to the large sample used in all data sets, it was not considered for judging model fit, although it was reported. Regarding the interpretation of the resulting effect sizes, the normative correlation guidelines suggested by Gignac and Szodorai (2016) were considered: 0.10, 0.20, and 0.30 indicate relatively small, typical, and relatively large, respectively. All analyses were performed using R packages (R Core Team 2020).

## 3. Results 

### 3.1. CFA Measurement Models 

Figure 1a,b displays the two measurement models tested in this step. As seen in Table 1, both CFA correlated-factor and bifactor models across the three samples fit the data adequately, with a slight advantage of the bifactor models. All factor loadings from the correlated-factor models were significantly different from zero across the three samples (*p* ≤ 0.001), as shown in Table 2. For each sample, the Table Reading subtest on the perceptual speed factor had the weakest loading (0.48–0.51), whereas the Verbal Analogies subtest on the verbal ability factor had the largest loading (0.91–0.94). With respect to intercorrelations among the five factors, Table 3 indicates that the weakest across the three samples were those between aviation acquired knowledge and quantitative ability (0.19–0.27), while the strongest were those between spatial ability and perceptual speed (0.67–0.76).

For the bifactor models, the standardized loading coefficients of the ten subtests on both the *g* factor and specific-ability factors are also presented in Table 2. Even in the presence of *g*, the five ability factors in the three samples remained clearly evident with significant loadings. Across samples, the factor that seemed most greatly influenced by the presence of *g* in the models was the spatial ability factor, as indicated by the weak loadings of its two indicators (0.20 to 0.30). The lowest loadings on the general factor were those produced by the Aviation Information subtest, one of the two indicators of the aviation-related acquired knowledge factor (0.05, *p* = 0.07 for the pilots’ sample; 0.17, *p* < 0.001 for the navigators’ sample; 0.29, *p* < 0.001 for the ABM sample). Overall, this initial phase of the study confirmed the soundness of the suggested five-factor model for aviation trainee cognitive abilities. 

### 3.2. Abilities–Performance Relationships 

**Figure 2:**
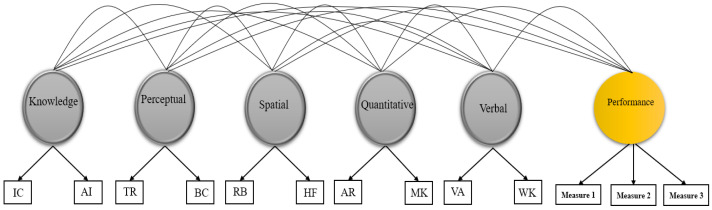
Representation of combined correlated-factor model containing cognitive abilities and performance measures. Note: VA = Verbal Analogies; WK = Word Knowledge; AR = Arithmetic Reasoning; MK = Math Knowledge; RB = Rotated Blocks; HF = Hidden Figures; TR = Table Reading; BC = Block Counting; IR = Instrument Comprehension; AI = Aviation Information. Performance presented here is latent, as is that modeled in pilot and navigator samples.

Combined correlated-factor models associating the five cognitive abilities with job performance criteria were then specified. Figure 2 presents an example of a tested model containing ability factors and one latent performance criterion. The CFA fit statistics of this model for the three samples were all acceptable, as presented in Table 1. The result of this analysis is presented in Table 4. All factors correlated substantially with the latent variable of pilots’ performance, with the exception of the verbal ability factor (*r* = −0.01, *p* > 0.10). The magnitudes of the significant relations ranged from 0.11 (quantitative ability) to 0.32 (aviation acquired knowledge). For navigators, the relations of cognitive abilities with latent performance outcomes were all significant with no exception, ranging from 0.13 (verbal ability) to 0.40 (spatial ability). For air battle managers, the correlations between the five abilities and the observed performance variable were also all significant, ranging between 0.19 (perceptual speed) and 0.32 (quantitative ability). This phase of analyses indicated that associations between cognitive abilities and job training performance were generally significant, although the pattern differed across occupations.

### 3.3. The Effects of g and Specific Abilities on Job Performance (Bifactor Model) 

**Figure 3:**
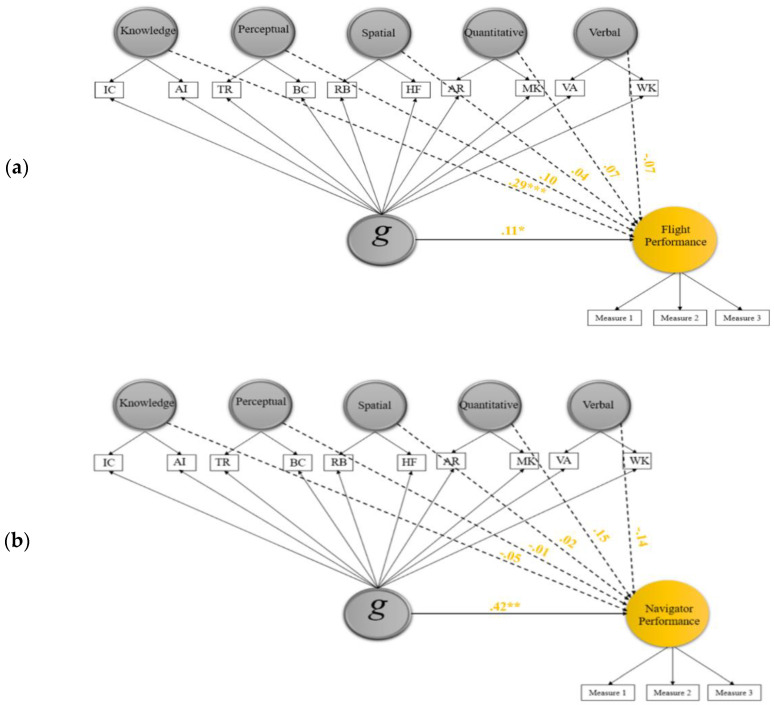

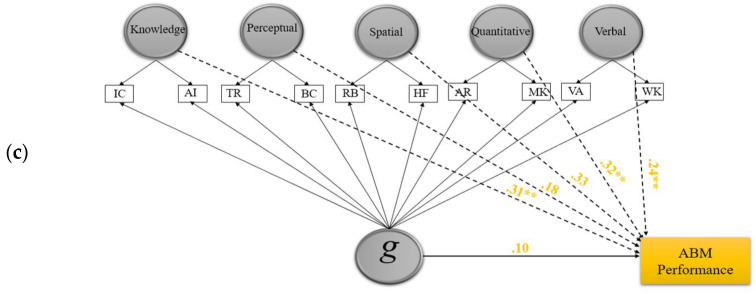
Bifactor SEM model for pilot (**a**), navigator (**b**), and air battle manager (**c**) data and the predictive validity estimates of general ability and specific abilities on job performance. Note: VA = Verbal Analogies; WK = Word Knowledge; AR = Arithmetic Reasoning; MK = Math Knowledge; RB = Rotated Blocks; HF = Hidden Figures; TR = Table Reading; BC = Block Counting; IR = Instrument Comprehension; AI = Aviation Information. Loadings of indicator pairs on their specific factor were constrained to be equal for model identification.

In this phase, a bifactor modeling approach was used to investigate the predictive value of *g* and five domain-specific abilities. Figure 3a–c display the structural models tested for pilot, navigator, and air battle manager data, respectively. As shown, the latent performance variables of pilots and navigators and the observed performance variable of air battle managers were regressed on the six abilities included in the models. Model fit statistics of the three predictive bifactor models are presented in Table 1. The models fit the three correlation matrices well (pilots: CFI = 0.94, RMSEA = 0.07, SRMR = 0.05; navigators: CFI = 0.95, RMSEA = 0.05, SRMR = 0.04; battle managers: CFI = 0.95, RMSEA = 0.07, SRMR = 0.05). 

Results presented in Table 5 and Figure 3a–c show interesting findings concerning the predictive relation of general ability and job performance. Among the three samples, the *g* effect was notably high only in the navigation sample, where its estimate was the only significant estimate among predictors in the model, with relatively high beta (β = 0.42, *p* < 0.01). Conversely, the *g* effect was small and not significant in the ABM sample (β = 0.10; *p* > 0.05), whereas three specific abilities emerged as strong predictors: quantitative ability, aviation acquired knowledge, and verbal ability (β = 0.32, 0.31, 0.24, respectively; *p* < 0.01). For the pilot sample, the *g* effect was rather small and barely reached the significance level (β = 0.11; *p* < 0.05), whilst aviation acquired knowledge, the only meaningful specific predictor in the model, predicted flight performance fairly strongly (β = 0.29, *p* < 0.001).

## 4. Discussion 

Intelligence researchers have long debated whether the general ability factor is the only factor that accounts for performance in cognitive tasks or if there might be other broad ability factors that explain some of the common variance in test scores (e.g., Agnello et al. 2015; Reeve and Bonaccio 2011). Another version of this debate is the debate among industrial/organizational (I/O) psychology researchers about whether it is general ability or narrower abilities that contribute most to the prediction of job and training performance (e.g., Hunter 1986; Kell and Lang 2017; Lang et al. 2010; Ones et al. 2017). The current study weighs in on this controversy by providing results that may be of mutual interest to intelligence and I/O psychology researchers using data from highly cognitively demanding occupations, where individual differences in job performance are linked to differences in cognitive abilities. Evidence from three aviation occupations was provided aboutthe predictive relations between cognitive abilities and job performance. Through the application of bifactor predictive models, results clarify the interplay of general and specific cognitive abilities in predicting the training performance of pilots, navigators, and air battle managers. 

The effect size of bifactor *g* was large in the navigator sample, small in the pilot sample, and negligible in the air battle manager sample. In contrast, the number of significant effects due to specific factors was none in the navigation sample, one in the flying sample, and three in the ABM sample. In the navigator sample, when *g* was modeled, the effect of specific abilities either declined or faded away, as compared to their significant relationships with performance criteria in the correlated-factor model. *g* was found to be the only noteworthy predictor for navigators’ performance, suggesting that the simple correlations of the five abilities with navigation performance were mostly due to their overlap with *g*. Navigation, like flying, is considered a complex class of jobs that requires high cognitive ability, even to undertake the training. In the old 16-subtest AFOQT (e.g., Carretta and Ree 1996), navigation applicants had to be qualified by an 11-subtest composite score (Navigator/Technical composite), as compared to an 8-subtest composite score (Pilot composite) for the qualification of pilot applicants. This gives an indication of the cognitively demanding nature of this job that may also explain the greater role of *g*, relative to specific abilities, in the prediction of trainees’ performance in navigation tasks. 

The pattern in the pilots’ sample comes in between the patterns noted for navigators and air battle managers, where aviation acquired knowledge, along with *g*, stayed significant and effective in the predictive model. Acquired aviation knowledge became a better predictor of flight performance after removing the general factor variance from its scale scores. The effect of this factor was estimated to be 0.29 versus 0.11 for the *g* factor. The higher effect of the aviation-related acquired knowledge factor in the pilot sample than in the other two samples may reflect the fact that the two indicators used to extract the factor include content more related to pilot jobs than any other jobs in the USAF. The predictive utility of tests measuring acquired knowledge for pilot performance has been documented in a number of meta-analyses (ALMamari and Traynor 2019, 2020; Hunter and Burke 1994; Martinussen 1996).

The strong relationship between the AFOQT construct of aviation acquired knowledge and pilot performance has been distinctly determined in ALMamari’s (2021) study. Using a similar modeling technique to that presented here (i.e., bifactor) and three pilot performance criteria, effect sizes of 0.43 and 0.12 were obtained for this construct when predicting “hands-on” flying performance at primary and advanced phases of training, respectively. For the academic performance criterion, acquired knowledge showed a weaker role (β = 0.08), although still noteworthy. Bifactor *g* related more strongly to academic performance (β = 0.24) and less strongly to hands-on pilot performance at the primary phase (β = 0.26) than acquired knowledge did. Perceptual speed ability demonstrated the highest predictive validity for hands-on pilot performance at the advanced phase. The remaining cognitive abilities (verbal, quantitative, and spatial) contributed trivially to pilot performance predictive models. 

Job knowledge test scores often demonstrate strong relationships with job performance (Hunter 1986; McDaniel et al. 1988). Hence, the comparative importance of this factor in the current finding may not be different from the trend. What makes the finding different, however, is the relatively large significant effect of this factor even with the presence of *g*, although it is common to hypothesize that job knowledge influences job performance indirectly through its relation with *g* (Ree et al. 1995; Schmidt et al. 1986). Interestingly, the knowledge-based tests can also be viewed as indicators of an applicant’s interest and motivation toward the job they are applying for (e.g., Kanfer and Ackerman 1989; Colquitt et al. 2000), and thus, it may be this interaction between the cognitive and non-cognitive aspects of the construct that makes this factor a robust predictor for pilot performance. 

Compared to flying and navigation performances, cognitive abilities’ predictive relations with air battle managers’ performance showed a distinct pattern that seemed somewhat unexpected. Due to the nature of the air battle manager performance measure as an average score of multiple written tests, the expectation was that this measure would relate more strongly to general ability than any specific ability due to its saturation with general academic and knowledge constructs. The influence of *g* on academic and achievement performance is a well-documented phenomenon (Gottfredson 2002; Gustafsson and Undheim 1996), especially when the performance is general in scope (Kahana et al. 2002) as is the case in air battle managers’ composite measure. However, contrary to expectations, quantitative ability, aviation acquired knowledge, and verbal ability were the three strongest predictors of air battle manager performance after removing the general factor variance in their latent scores. Thus, the current findings that seem in contrast with the majority of research supporting a dominant role of *g* over any specific abilities in the prediction of academic performance remain to be explained. 

One possible reason for the significance of specific abilities and non-significance of *g* as predictors of air battle manager performance is the way that air battle manager performance was modeled in this study. Due to the existence of only one performance measure for the air battle manager performance, it was modeled as an observed variable indicated by one dimension of performance more related to academic achievement, rather than modeling it as a latent variable indicated by multiple measures of different performance dimensions. Including scores from multiple dimensions of air battle manager performance may make the construct more suitable to be predicted by a general predictor such as *g* (e.g., Ones and Viswesvaran 1996). Additionally, performance measures of pilots and navigators in this study relied primarily on ratings of hands-on job samples, while that of air battle managers was mostly academic, which may not correspond well to our operationalization of *g* that includes spatial ability and perceptual speed, which were probably not sampled in the conventional academic test items. 

Moreover, according to the job complexity hypothesis, a highly complex job requires more general ability, and a less complex job requires only specific abilities (Gottfredson 1997; Hunter et al. 1990; Murphy 1989). Thus, the air battle manager performance in this study may have been represented by a less complex dimension in the wide criterion space of the ABM job, while the performance of pilots and navigators was represented by a global score with overlapping dimensions and constructs, most of which were practical in their essence. Furthermore, an air battle manager’s job is generally less complex than pilot and navigator jobs (e.g., Fowley 2016; Rhone 2008), with a lower minimum qualifying score (Carretta 2008), and thus, also based on the job complexity proposition, a lesser role for g might be expected. Last, it is expected that the courses taught in a technical program for training air battle managers are also of technical scope and tend to target narrower knowledge and skills. According to the ability–criterion compatibility principle (Schneider and Newman 2015), such a specific-ability-oriented criterion score is best predicted by a specific-ability-oriented score.

All in all, despite pre-existing overwhelming evidence of the supremacy of the general factor as the best stand-alone predictor of job and training performance (Ones et al. 2017; Ree and Earles 1992; Schmidt and Hunter 2004), the present study provides support for crucial predictive roles for some specific abilities that contribute uniquely to performance outcomes. A strong predictive role for some specific abilities (relative to g) for job and training performance has also been found in some recent investigations (e.g., Coyle 2018; Lang and Kell 2020; Lang et al. 2010; Ziegler et al. 2011), implying that this conclusion may hold across a wider range of occupations. In our view, a next step should be to synthesize this accumulating evidence to characterize more systematically which specific abilities tend to predict job performance outcomes and net g, which performance outcomes, and in what types of job roles. Following Brunswik and Krumm et al. (2014), we might then ask, in each study that has found stand-alone predictive value of specific factors, what were the indicator components of g, their analytic weights or loadings, and the indicator components of any specific factors and their weights, and to what extent were they aligned with the indicators of successful job performance? 

## 5. Implication for Selection and Training 

For psychometricians developing or maintaining selection test batteries for aviation professions, it is recommended that they increase the attention given to the construct of aviation acquired knowledge in the selection process as it is shown to be a strong orthogonal predictor of air battle manager and pilot (although not navigator) performance outcomes. Flight programs outside the USAF that have traditionally based student admission decisions primarily on educational achievement or aptitude scores, such as GPA, SAT, or ACT scores, might consider introducing more standardized evidence of applicants’ aviation acquired knowledge into their selection processes or allowing students who have demonstrated a certain baseline level of knowledge to forego introductory courses and proceed to more advanced levels of training. Recognizing that the nature of military and civilian aviation occupations is changing with increasing automation, we expect attention may also be needed to developing or maintaining relevant aviation-acquired-knowledge test tasks. 

If a goal is to optimize selection and prediction, our results also may suggest that the latent cognitive constructs underlying AFOQT total scores should not be given equal weight in generating scores to evaluate an applicant’s readiness for training or in predicting their future performance outcomes (and that the scores weighting for these two predictive purposes should likely be different). Presently, the AFOQT developers compute six composite scores from ten AFOQT subtests (Form T). The construction of these composites is mostly conceptual groupings of the subtests, rather than factor-based. Factor scores may provide more accurate representations of the relations between CHC broad ability constructs and achievement or performance outcomes than total scores created by summing subtest scores do (Benson et al. 2016). Hence, future scoring development of the AFOQT might include factor scores to overcome problems typically associated with the more common practice of subtest analysis. 

Finally, specific recommendations from a selection perspective for youth program administrators promoting aviation professions as a potential career are to (a) emphasize the importance of cognitive abilities as selection criteria for flight programs and that these abilities can be developed by practice, (b) determine specific cognitive abilities that serve as significant predictors of aviation performance among young people, (c) familiarize participants with common ability test batteries used in selection, and (d) provide opportunities for training directed toward the most important cognitive abilities for aviation professionals.

## 6. Limitations and Future Research 

In this study, the role of general ability and domain-specific abilities as predictors for job performance was examined using data for three aviation-related occupations. The main focus was five ability factors, along with psychometric *g*, that can be extracted from AFOQT subtest scores. The results of the present study show that the predictive relations differed across three professional aviation occupations. Although the cognitive testing applied on the three samples was similar, the performance measures differed to some extent, especially that of the ABM sample. The breadth of performance measures used in each sample (e.g., general or specific), the varying modeling approach (e.g., latent or observed), and the constraints we imposed to identify the predictive bifactor model (e.g., Eid et al. 2018; Zhang et al. 2021) could have had some effect on the results. To allow a better comparison, future studies should attempt to obtain comparable performance measures across occupations, such as academic performance of the training program or actual hands-on experience of the job sample. The ability–performance relationship was investigated in this study without controlling for any potential covariates that may influence the predictive relations. Future studies that aim to establish the validity of cognitive abilities for job performance could add to the predictive models potential moderator variables, such as gender or ethnicity group, if such moderators were suggested by previous empirical findings. Future studies could expand the scope and assess the predictive role of other cognitive functions obtained from different test batteries (e.g., memory, multitasking, reaction time) for professional flight occupations. Due to the limitation of cross-sectional data and the likely influence of between-group sampling variability (Little 2013), future studies may also attempt a longitudinal design for tracking the changes of predictive relations through different phases of training, with some control of previous levels of variables. 

The modeling technique applied in the present study was based primarily on a bifactor model, which has some inherent limitations (Eid et al. 2018; Reise 2012; Zhang et al. 2021). For example, the factors in a bifactor model, both the general factor and grouping factors, are restricted to being uncorrelated. In addition, each indicator in a bifactor model is allowed to load onto the general factor and to only one grouping factor. Due to the known intercorrelations between cognitive data, these assumptions may seem unrealistic, where group factors are conceptually related, or an indicator can mark more than one construct. For example, perceptual speed and spatial ability factors are expected to share some common variance that is attributable to the general factor (e.g., Barron and Rose 2013), but each was marked with a separate set of indicators. Thus, it would be useful to attempt different approaches with other analytic procedures to give the results further credibility. Examples of such approaches that have shown to be effective in separating the effects of predictors on a criterion include relative weight analysis (Johnson 2000), dominance analysis (Azen and Budescu 2003), and the non-g residuals of tests derived from a higher-order factor structure (Coyle 2018). Replicating the results of the current study using some of these methods can give further confidence in the results. Finally, because this study attempted to provide a view of ability–performance relationships different from the conventional view that relies on corrected data, the findings are likely to be an underestimation of the true effects of cognitive abilities on job performance measures. Using correlational data that are corrected for attenuation (e.g., range restriction) may show a different or substantially similar pattern of predictive relations (Aguinis et al. 2011), although those data transformation techniques also have limitations (LeBreton et al. 2014). Given the restrictive samples used in this study due to the strict selection procedures for USAF officer candidates, especially those qualified for aviation jobs, generalization of current findings to less restricted samples from similar occupations (e.g., civil airline pilots) needs to be made with caution. 

## 7. Conclusions 

The interplay of cognitive abilities as predictors of job performance in three aviation-related occupations showed a pattern that is different from the body of evidence pertaining to the influence of general ability on performance criteria. Removing the systematic variance associated with the general factor produces larger effects for some specific ability factors predicting job performance (e.g., aviation acquired knowledge in the pilot sample) while diminishing the effects of some other specific factors (e.g., spatial ability in the navigation sample). The significant effects revealed in the current study for some cognitive abilities have practical importance. Based on the results of bifactor modeling, the general ability in this study is a strong predictor of navigation performance, a weak predictor of pilot performance, and a negligible predictor of air battle manager performance. The approach to cognitive modeling utilized in this study opens an opportunity for future research seeking to enhance the predictive validity of ability constructs for job performance.

## Figures and Tables

**Table 1:** Fit statistics of all models tested in the study.

	Model	χ^2^	df	CFI	RMSEA	SRMR
Pilots	CFA Correlated-factor	397.29	30	0.91	0.08	0.07
	CFA Bifactor	333.25	30	0.92	0.07	0.05
	Combined Correlated-factor	558.47	55	0.93	0.07	0.06
	SEM Bifactor	494.52	54	0.94	0.07	0.05
Navigators	CFA Correlated-factor	158.22	30	0.93	0.07	0.06
	CFA Bifactor	135.27	30	0.95	0.06	0.05
	Combined Correlated-factor	186.63	55	0.94	0.05	0.05
	SEM Bifactor	162.03	54	0.95	0.05	0.04
Air Battle	CFA Correlated-factor	138.26	30	0.94	0.07	0.07
Managers	CFA Bifactor	121.74	30	0.95	0.07	0.05
	Combined Correlated-factor	158.68	35	0.94	0.07	0.06
	SEM Bifactor	141.22	34	0.95	0.07	0.05

**Table 2:** Factor loadings from correlated-factor and bifactor models.

Sample	Model	Factor	Verbal		Quantitative		Spatial		Perceptual		Knowledge	
			VA	WK	AR	MK	RB	HF	TR	BC	IC	AI
Pilots	Correlated-factor		0.94	0.60	0.82	0.69	0.51	0.56	0.48	0.75	0.62	0.64
	Bifactor	Specific	0.80	0.50	0.51	0.40	0.27	0.30	0.31	0.49	0.59	0.65
		General	0.49	0.38	0.61	0.62	0.51	0.41	0.40	0.55	0.38	0.05
Navigators	Correlated-factor		0.91	0.60	0.86	0.65	0.51	0.54	0.48	0.79	0.67	0.64
	Bifactor	Specific	0.78	0.50	0.65	0.48	0.20	0.23	0.31	0.51	0.58	0.59
		General	0.50	0.37	0.55	0.47	0.54	0.43	0.38	0.59	0.46	0.17
Air Battle Managers	Correlated-factor		0.91	0.70	0.81	0.76	0.69	0.65	0.51	0.87	0.67	0.69
	Bifactor	Specific	0.78	0.59	0.59	0.60	0.27	0.26	0.34	0.61	0.52	0.59
		General	0.48	0.39	0.58	0.43	0.63	0.60	0.43	0.60	0.56	0.29

Note. All loadings were significant at *p* < 0.001, except for the Aviation Information subtest on *g* factor in the pilot sample. (*p* = 0.07).

**Table 3:** Summary for the factor intercorrelations resulting from the CFA correlated-factor models.

		**Verbal**	**Quantitative**	**Spatial**	**Perceptual**	**Knowledge**
Pilots	Verbal	1				
	Quantitative	0.55	1			
	Spatial	0.42	0.59	1		
	Perceptual	0.28	0.58	0.74	1	
	Knowledge	0.16	0.19	0.45	0.29	1
		**Verbal**	**Quantitative**	**Spatial**	**Perceptual**	**Knowledge**
Navigators	Verbal	1				
	Quantitative	0.50	1			
	Spatial	0.46	0.54	1		
	Perceptual	0.34	0.47	0.76	1	
	Knowledge	0.29	0.26	0.55	0.36	1
		**Verbal**	**Quantitative**	**Spatial**	**Perceptual**	**Knowledge**
Air Battle	Verbal	1				
Managers	Quantitative	0.52	1			
	Spatial	0.46	0.55	1		
	Perceptual	0.31	0.50	0.67	1	
	Knowledge	0.32	0.27	0.67	0.42	1

Note. All correlations were significant at *p* < 0.001.

**Table 4:** Correlations between cognitive abilities and job performance.

	Flying	Navigation	Air Battle Management
Verbal Ability	−0.01 ^ns^	0.13 **	0.29 ***
Quantitative Ability	0.11 ***	0.37 ***	0.32 ***
Spatial Ability	0.15 ***	0.40 ***	0.22 ***
Perceptual Speed	0.17 ***	0.32 ***	0.19 ***
Acquired Knowledge	0.32 ***	0.16 ***	0.29 ***

Note. Gray color indicates negative estimate or nonsignificant positive estimate (*p* > 0.05). Model fit was as follows. ns, *p* < 0.10. ** *p* < 0.01. *** *p* < 0.001.

**Table 5:** Prediction of job performance by general ability and specific abilities via bifactor models.

	Flying	Navigation	Air Battle Management
Verbal Ability	−0.07 ^ns^	−0.14 ^ns^	0.24 **
Quantitative Ability	0.07 ^ns^	0.15 ^ns^	0.32 **
Spatial Ability	0.04 ^ns^	0.02 ^ns^	0.33 ^ns^
Perceptual Speed	0.10 ^ns^	−0.01 ^ns^	0.18 ^ns^
Acquired Knowledge	0.29 ***	−0.05 ^ns^	0.31 **
General Ability (g)	0.11 *	0.42 **	0.10 ^ns^

Note. Gray color indicates negative estimate or nonsignificant positive estimate (*p* > 0.05). ns, *p* < 0.10. * *p* < 0.05. ** *p* < 0.01. *** *p* < 0.001.

## Data Availability

Data sharing not applicable—no new data generated. See Appendix A for the test score correlation matrices analyzed.

## Appendix A

**Table A1:** Correlations among variables for pilot, navigator, and air battle manager samples.

**Pilot Sample**													
	**VA**	**AR**	**WK**	**MK**	**IC**	**BC**	**TR**	**AI**	**RB**	**HF**	**C1**	**C2**	**C3**
**VA**	1												
**AR**	0.39	1											
**WK**	0.59	0.33	1										
**MK**	0.40	0.58	0.33	1									
**IC**	0.13	0.19	0.11	0.15	1								
**BC**	0.20	0.32	0.13	0.30	0.27	1							
**TR**	0.11	0.29	0.09	0.28	0.20	0.37	1						
**AI**	0.05	0.05	0.12	−0.06	0.40	0.00	0.00	1					
**RB**	0.22	0.27	0.14	0.29	0.29	0.35	0.15	0.09	1				
**HF**	0.20	0.20	0.14	0.24	0.20	0.27	0.18	0.04	0.29	1			
**C1**	0.00	0.11	−0.02	0.04	0.23	0.10	0.15	0.18	0.10	0.06	1		
**C2**	0.00	0.14	−0.01	0.06	0.28	0.09	0.12	0.27	0.10	0.04	0.68	1	
**C3**	0.00	0.08	−0.02	0.03	0.17	0.11	0.13	0.12	0.06	0.07	0.76	0.49	1
***M***	15.32	13.27	16.08	16.57	11.26	12.71	29.8	10.75	8.93	10.43	0.79	72.04	81.59
***SD***	3.22	3.76	5.14	4.73	4.4	3.74	6	4	2.85	2.5	0.4	13.02	7.4
**Navigator Sample**													
	**VA**	**AR**	**WK**	**MK**	**IC**	**BC**	**TR**	**AI**	**RB**	**HF**	**C1**	**C2**	**C3**
**VA**	1												
**AR**	0.36	1											
**WK**	0.57	0.35	1										
**MK**	0.34	0.57	0.29	1									
**IC**	0.20	0.20	0.15	0.18	1								
**BC**	0.25	0.30	0.16	0.22	0.29	1							
**TR**	0.14	0.26	0.15	0.22	0.18	0.38	1						
**AI**	0.12	0.07	0.17	0.04	0.42	0.07	0.00	1					
**RB**	0.24	0.25	0.12	0.21	0.32	0.37	0.18	0.15	1				
**HF**	0.21	0.23	0.13	0.20	0.19	0.28	0.15	0.07	0.28	1			
**C1**	0.09	0.24	0.05	0.20	0.12	0.17	0.17	0.04	0.19	0.12	1		
**C2**	0.05	0.10	0.04	0.13	0.05	0.09	0.14	0.00	0.07	0.05	0.33	1	
**C3**	0.06	0.15	0.05	0.13	0.05	0.09	0.13	0.04	0.14	0.12	0.31	0.17	1
***M***	15.10	13.18	15.04	16.61	14.09	13.05	30.50	14.04	9.34	10.49	0.84	87.80	85.6
***SD***	3.23	3.66	5.09	5.00	4.13	3.58	5.94	4.05	2.81	2.48	0.36	13.33	15.4
**Air Battle Manager Sample**													
	**VA**	**AR**	**WK**	**MK**	**IC**	**BC**	**TR**	**AI**	**RB**	**HF**	**C1**		
**VA**	1												
**AR**	0.43	1											
**WK**	0.65	0.35	1										
**MK**	0.29	0.61	0.24	1									
**IC**	0.22	0.24	0.18	0.17	1								
**BC**	0.24	0.34	0.21	0.29	0.35	1							
**TR**	0.15	0.35	0.10	0.28	0.24	0.47	1						
**AI**	0.17	0.13	0.21	0.02	0.47	0.13	0.11	1					
**RB**	0.29	0.34	0.22	0.25	0.41	0.38	0.21	0.25	1				
**HF**	0.27	0.31	0.20	0.24	0.38	0.37	0.27	0.18	0.44	1			
**C1**	0.25	0.27	0.27	0.22	0.18	0.14	0.20	0.22	0.18	0.12	1		
***M***	17.58	15.39	16.49	16.22	13.11	13.68	29.03	9.26	8.99	10.26	94.43		
***SD***	3.55	4.87	4.68	4.8	4.85	3.62	6.42	4.22	2.98	3.17	2.47		

Note. VA = Verbal Analogies; AR = Arithmetic Reasoning; WK = Word Knowledge; MK = Math Knowledge; IC = Instrument Comprehension; BC = Block Counting; TR = Table Reading; AI = Aviation Information; RB = Rotated Blocks; HF = Hidden Figures; C = Criterion; M = Mean; SD = Standard Deviation; for pilot sample: C1 = Pass/Fail training, C2 = Phase 2 average rating, C3 = Phase 3 average rating; for navigator sample: C1 = Pass/Fail training, C2 = day check flight average rating, C3 = night check flight average rating; for ABM sample: C1 = average final score on several written tests. Correlation matrices were obtained from Olea and Ree (1994) for pilot and navigator samples and from Carretta (2008) for air battle management sample.
